# Peri-articular Dextrose Prolotherapy: Investigating the Effect of Injection Site on Knee Osteoarthritis Pain: A Double-Blind, Randomized, Clinical Trial

**DOI:** 10.5812/aapm-140966

**Published:** 2024-03-27

**Authors:** Shahrokh Ebnerasooli, Arash Barghi, Karim Nasseri, Nasrin Moghimi

**Affiliations:** 1 Department of Anesthesiology, Faculty of Medicine, Kurdistan University of Medical Sciences, Sanandaj, Iran; 2 Department of Anesthesiology, Kurdestan University of Medical Sciences, Sanandaj, Iran; 3 Department of Anesthesiology, Ardabil University of Medical Sciences, Ardabil, Iran; 4 Department of Internal Medicine, Kurdestan University of Medical Sciences, Sanandaj, Iran

**Keywords:** Dextrose Prolotherapy, Knee Osteoarthritis, Pain, Peri-articular, WOMAC

## Abstract

**Background:**

Osteoarthritis (OA) is a chronic health condition that affects millions of people worldwide. It not only causes pain and physical limitations but also impacts mental health, sleep, work participation, and even mortality. Peri-articular dextrose prolotherapy has been shown to reduce knee osteoarthritis pain; however, the effect of injection sites on its effectiveness is not clear.

**Objectives:**

This study aimed to investigate the effect of injection points on pain intensity, joint stiffness, and physical activity in patients with knee osteoarthritis who underwent peri-articular dextrose prolotherapy.

**Methods:**

This double-blind clinical trial involved 26 patients with grade 2 and 3 bilateral knee osteoarthritis. Three times every one week, dextrose and lidocaine were injected as interventions. Injection sites were positioned within acupuncture points on one knee, but were relocated by 1.5 centimeters to the medial side of the same acupuncture points on the other knee. Pain intensity, joint stiffness, and physical activity were evaluated using the Visual Analog Pain Scale (VAS) and the Persian version of the Western Ontario and McMaster Universities Osteoarthritis Index (WOMAC) before the intervention and at each follow-up visit at 1 and 2 months post-injection.

**Results:**

Pain intensity score, joint stiffness, physical performance, and WOMAC were significantly decreased one and two months after the intervention in both groups (P = 0.0001). The improvement in the patients of both groups was similar, and the two study groups did not have a statistically significant difference in terms of study outcomes (P = 0.37).

**Conclusions:**

Prolotherapy with dextrose is an effective treatment for knee osteoarthritis.

## 1. Background

Osteoarthritis (OA) is a prevalent chronic health condition that significantly impacts various aspects of individuals' lives (1). It affects millions of people worldwide, with knee osteoarthritis being particularly influenced by factors such as obesity and age (2-4). In Kurdistan state, the incidence of knee OA was 18.8%, compared to 15.3% in Iran (5). Managing OA involves non-pharmacological interventions alongside pharmacological treatments (2, 6).

The initial treatment approach for knee osteoarthritis should consist of non-pharmacological therapies, including weight management, physical exercise, systemic non-steroidal and steroidal anti-inflammatory drugs, opioids, platelet-rich plasma injections into the joint, placebo treatments, corticosteroid injections into the joint, viscosupplementation within the joint, and surgical intervention (2). Among the recommended treatment modalities is dextrose prolotherapy, which involves the injection of materials to initiate repair in the joint (7-9).

The most frequently utilized prolotherapy substance in clinical settings is dextrose, typically administered in concentrations ranging from 12.5% to 25% (10). Dextrose is regarded as an outstanding proliferant due to its solubility in water, its status as a natural component of blood chemistry, and its ability to be safely injected into multiple areas in substantial amounts. The mechanism of action of prolotherapy is not fully comprehended (10).

A single study has shown that stimulating acupuncture sites with needles may improve pain relief, lessen disability, and reduce the need for analgesic and anti-inflammatory drugs. This positive outcome is attributed to the release of endorphins and enkephalins (11). In a randomized clinical trial, injection of dextrose in acupuncture points compared to injection in the intra-articular space had similar results, and injection in both of these places led to a reduction in pain and improved performance of the patients (9).

## 2. Objectives

Since it is unclear whether the reduction of pain and disability caused by the peri-articular injection of dextrose in the acupuncture points around the knee is related to the inflammatory and proliferative effects of dextrose or the stimulation of the acupuncture points caused by the insertion of the needle and injected dextrose, this study aims to compare the differences in treatment results between peri-articular dextrose prolotherapy applied at relevant acupuncture sites and non-matching acupuncture points around the knee.

## 3. Methods

This study was a double-blind clinical trial conducted on 26 patients with grade 2 and 3 bilateral knee osteoarthritis. Inclusion criteria for the study required patients to meet at least three of six American College of Rheumatology (ACR) criteria, including age over 50 years, morning stiffness less than 30 minutes, crepitation in active knee movements, bone tenderness, bone enlargement, and lack of heat when touching the joint. Exclusion criteria included: The injection of steroid drugs in the last 2 months, diabetes mellitus, candidates for knee arthroplasty, previous injection treatment with dextrose, knee infection in the last three months, knee inflammation, history of drug abuse, history of inflammatory joint diseases, infectious arthritis, joint dysplasia, congenital malformation, crystalopathy, post-traumatic arthritis, malignancy, vascular necrosis, and Body Mass Index (BMI) > 30.

### 3.1. Sample Size and Sampling

To determine the appropriate sample size for our study, we utilized an estimation method to compare the difference between two averages. We calculated the required sample size to be 26 people based on the difference in pain ratings between the two groups and a 95% confidence level with a 0.5 standard deviation. This equates to 52 knees evaluated, with each person having two knees assessed.

To minimize any potential bias, patients were randomly allocated into groups A and B. For the A group, the right knee was designated as the treatment knee and received an injection in the acupuncture points, while the left knee served as the control and received an injection in points located two centimeters medial to the acupuncture points. The opposite was done for the B group.

### 3.2. Study Design

After obtaining informed consent and approval from the ethics committee, the patients were randomly divided into two groups, odd group (A) and even group (B), based on the table of numbers. Patients in the A group were injected with hypertonic dextrose at the acupuncture sites on the right knee and 1.5 cm medial to the acupuncture points on the left knee. In the B group, patients received hypertonic dextrose injections at opposite points in the same knees.

### 3.3. Intervention

To prepare for the study, all analgesic drugs were stopped 48 hours prior, except for acetaminophen, which was taken orally every 8 hours starting 6 hours before the injection and continuing for 48 hours. Each patient in both groups received an intervention consisting of 8 mL of 10% dextrose and 2 mL of 2% lidocaine, with 2.5 mL of the drug solution injected at each of the 4 points using a 24 G needle. Three shots were administered, separated by one week. The acupuncture points injected into one knee were the same ones utilized in the earlier Rezasoltani et al. (9) study, but the injection sites in the other knee were moved 1.5 cm to the medial side of the identical acupuncture points. Blinding was conducted so that the research associate responsible for evaluating the results and the patients themselves were unaware of the grouping of the patients and the difference between the injection points in the two groups.

Patients were allowed to take 325 mg of oral acetaminophen three times a day during the study in case of pain. Pain and disability were evaluated using Western Ontario and McMaster Universities Osteoarthritis Index (WOMAC) before the intervention (zero time) and at each follow-up visit at 1 and 2 months post-injection.

### 3.4. Outcome Measurements

The study had two main outcomes: Pain and WOMAC criteria. Pain was measured using Visual Analog Pain Scale (VAS) with a range of 0 - 10. The Persian version of the WOMAC (12) was used to evaluate the outcome of the intervention at each follow-up visit. Twenty-four items were distributed across three subscales: Physical function, rigidity, and pain. The pain assessment comprised five inquiries encompassing different positions, such as walking, stair usage, bed transitions, sitting, standing, and other similar activities. Stiffness consisted of two questions at two times: After waking up and at the end of the day.

The physical activity section of the WOMAC questionnaire included 17 questions about various activities, such as using stairs, getting up, standing, bending, walking, entering and exiting a car, shopping, donning and doffing socks, rising from bed, turning over in bed, using the bathroom, and sitting down, toileting, heavy housework, and light housework. The scoring scale in the WOMAC questionnaire was evaluated as follows: 0 = none, 1 = little, 2 = moderate, 3 = very difficult, and 4 = very difficult.

To evaluate the effectiveness of the intervention, the results of the VAS score and WOMAC questionnaires were assessed at three time intervals: Before the intervention, one month after the last injection, and two months after the last injection. A colleague who was unaware of the grouping of the patients' knees and the injection sites around the knees interviewed the patients.

In addition to the primary outcome, the study collected demographic information about the patients, such as age, degree of osteoarthritis, and BMI, as well as any injection complications, such as pain, redness, itching, and infection. In addition to the primary outcome of the study, demographic information of the patients (age, sex, degree of osteoarthritis, BMI) and injection complications (pain, redness, itching, and infection) were evaluated and recorded.

### 3.5. Statistical Analysis

The collected data were analyzed using SPSS software (Version 18 for Windows. SPSS, Chicago, IL, USA). Descriptive statistics, such as the mean, standard deviation, frequency, and relative frequency, were employed to summarize the data. The normality of dependent quantitative variables was evaluated using the Kolmogorov-Smirnov test. Fisher's exact test, independent *t*-tests, and chi-square tests were employed to examine the correlations between the variables. Additionally, throughout the treatment period, scores in two groups were compared using a one-way repeated measures ANOVA. A P-value < 0.05 was considered statistically significant.

## 4. Results

**Figure 1.**
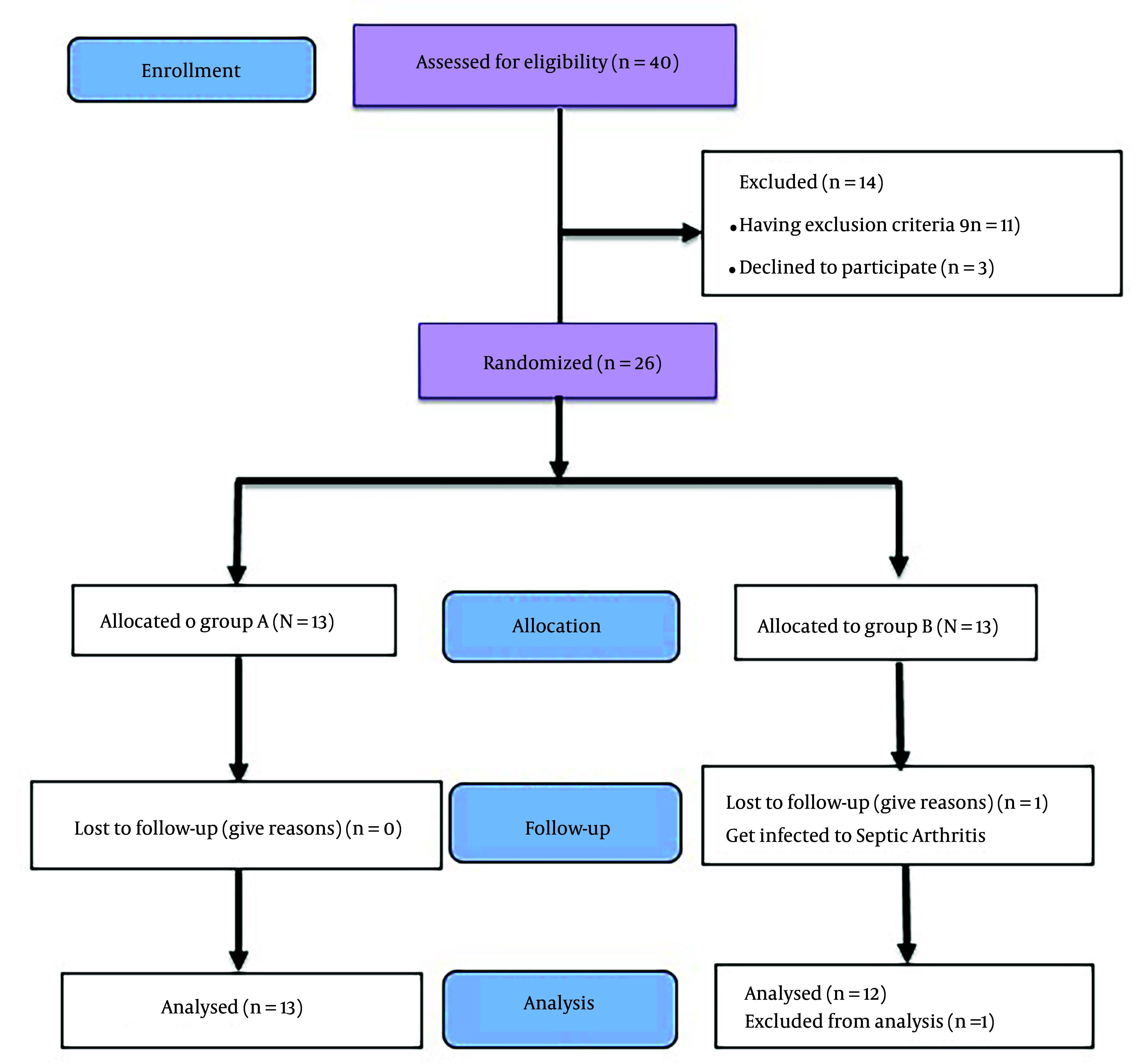
Flow diagram

Forty patients were selected to enter this study. Fourteen patients were excluded from the study before the start of the intervention. Eleven of them had exclusion criteria, while 3 declined to participate. Ultimately, 26 patients were randomly divided into two groups, A and B, and underwent intervention. One person in group B was excluded from the study because of infectious arthritis a few days after the first injection. Finally, 25 patients entered the analysis phase of this study (Figure 1). 

Patients in both groups (A and B) showed no significant differences regarding demographic data including age (61.5 ± 6.1 versus 58.1 ± 5.9), sex, BMI (27.1 ± 1.6 versus 28.2 ± 2.2), and osteoarthritis grade (Tables 1 and 2). 

**Table 1.** Comparison of Age, Body Mass Index, Scores of Pain Intensity, Joint Stiffness, and Physical Function

Variables	Mean ± SD	T	P-Value ^a^
**Age**		1.65	0.06
A	61.5 ± 6.1		
B	58.1 ± 5.9		
**Body mass index**		0.97	0.63
A	27.5 ± 1.6		
B	28.2 ± 2.2		
**Before intervention**			
Pain (VAS score)		0.1	0.99
A	14.2 ± 2.2		
B	14.2 ± 2.2		
Joint stiffness		0.6	0.57
A	6.2 ± 0.6		
B	6.2 ± 0.7		
Physical function		0.1	0.99
A	45.9 ± 5.9		
B	45.9 ± 5.9		
WOMAC		0.12	0.9
A	66.3 ± 7.8		
B	66.3 ± 7.7		
**1 month after intervention**			
Pain (VAS score)		1.8	0/08
A	10.5 ± 1.6		
B	10.6 ± 1.5		
Joint stiffness		1.4	0.16
A	4.6 ± 0.5		
B	4.7 ± 0.6		
Physical function		0.4	0.66
A	36.9 ± 4.8		
B	36.8 ± 5.0		
WOMAC		0.15	0.88
A	52.0 ± 5.8		
B	52.0 ± 6.0		
**2 months after intervention**			
Pain (VAS score)		0.3	0.75
A	6.6 ± 1.0		
B	6.5 ± 0.9		
Joint stiffness		0.1	0.99
A	2.9 ± 0.4		
B	2.9 ± 0.5		
Physical function		1.2	0.23
A	27.1 ± 4.6		
B	26.4 ± 4.0		
WOMAC		0.3	0.76
A	35.7 ±4.5		
B	35.8 ±4.9		

^a^ Groups are compared using the independent *t*-test.

**Table 2.** Comparison of Sex and Osteoarthritis Grade Between Two Study Groups

Variables	Group A ^a^	Group B ^a^	P-Value ^b^
**Osteoarthritis grade**			
Right knee			0.67
Grade 2	3 (23.1)	4 (33.3)	
Grade 3	10 (76.9)	8 (66.7)	
Left knee			0.41
Grade 2	3 (23.1)	5 (41.7)	
Grade 3	10 (76.9)	7 (58.3)	
**Sex**			0.32
Male	4 (30.8)	1 (8.3)	
Female	9 (69.2)	11(91.7)	

^a^ Values are expressed as No. (%).

^b^Groups are compared using the Fisher's exact test.

There were no significant differences between the two study groups regarding VAS score and the three WOMAC indexes (pain intensity, joint stiffness, and physical function) before the intervention, one month, and two months after the intervention (Table 1). 

**Figure 2.**
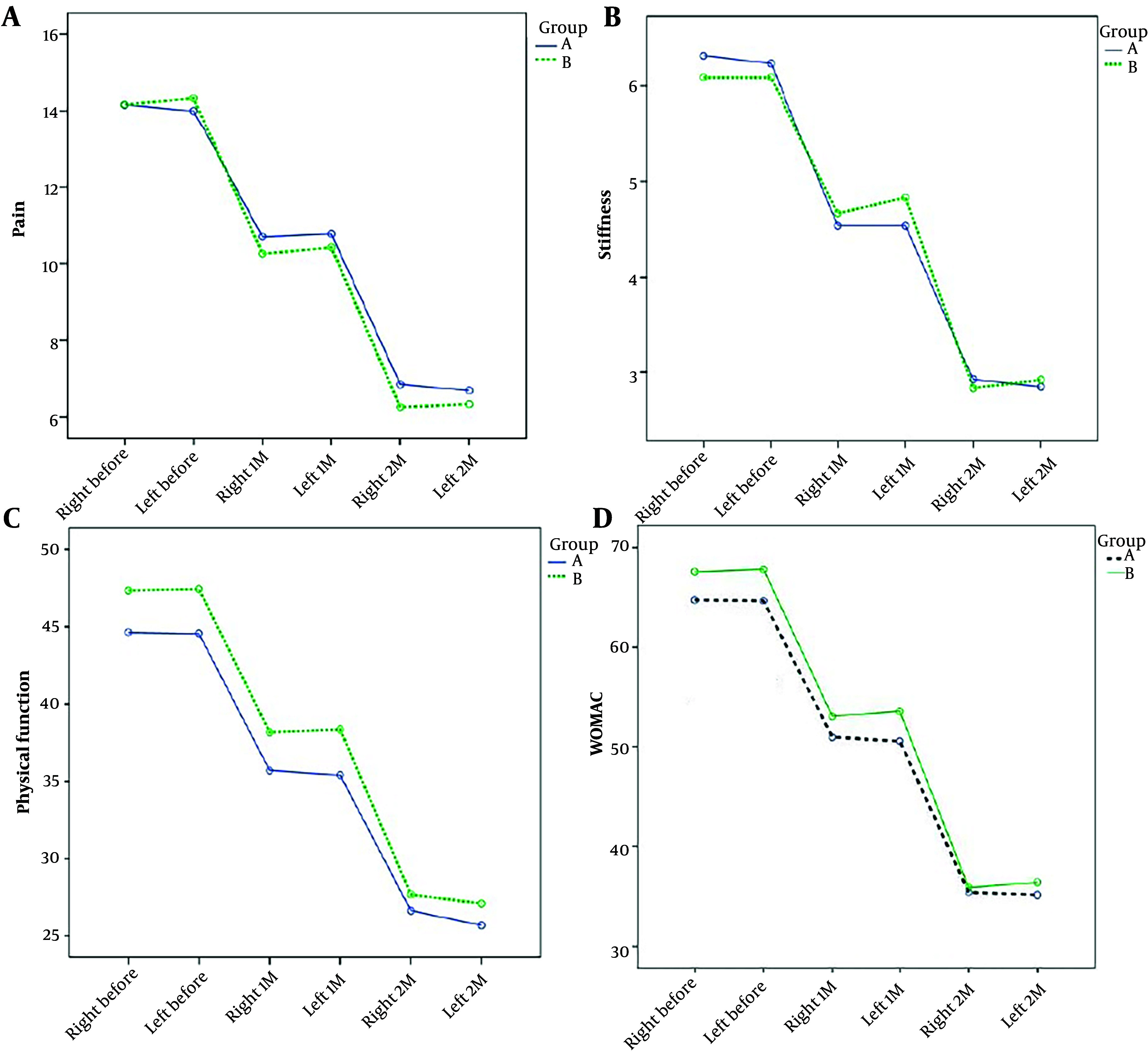
Comparison of the average knee pain score, average knee joint stiffness score, average physical function score, and total Western Ontario and McMaster Universities Osteoarthritis Index (WOMAC) score in two groups during the study period

Based on a repeated data analysis of variance test, the average knee pain score, average knee joint stiffness score, average physical function score, and overall WOMAC score in the two groups decreased significantly across the three phases of assessment (P = 0.0001). However, no statistically significant difference was observed between the two groups regarding the average knee pain score (P = 0.67), average knee joint stiffness score (P = 0.97), average physical function score (P = 0.24), and total WOMAC score (P = 0.37) (Table 3 and Figure 2). 

**Table 3.** Comparison of the Average Knee Pain Score, Average Knee Joint Stiffness Score, Average Physical Function Score, and Total Western Ontario and McMaster Universities Osteoarthritis Index Score in Two Groups During the Study Period

Variables and Groups	Mean ± SD	F	P-Value ^a^
**VAS knee pain**		1334.4	0.0001
Right knee before intervention			
A	14.2 ± 1.5		
B	14.2 ± 2.9		
Left knee before intervention			
A	14.0 ± 1.7		
B	14.3 ± 2.8		
Right knee 1 month after intervention			
A	10.7 ± 0.9		
B	10.3 ± 2.1		
Left knee 1 month after intervention			
A	10.8 ± 0.9		
B	10.4 ± 2.0		
Right knee 2 months after intervention			
A	6.8 ± 0.7		
B	6.2 ± 1.2		
Left knee 2 months after intervention			
A	6.7 ± 0.5		
B	6.3 ± 1.3		
**Knee joint stiffness**		1972.2	0.0001
Right knee before intervention			
A	6.3 ± 0.7		
B	6.1 ± 0.8		
Left knee before intervention			
A	6.2 ± 0.8		
B	6.1 ± 0.8		
Right knee 1 month after intervention			
A	4.5 ± 0.5		
B	4.7 ± 0.5		
Left knee 1 month after intervention			
A	4.5 ± 0.5		
B	4.8 ± 0.6		
Right knee 2 months after intervention			
A	2.9 ± 0.5		
B	2.8 ± 0.4		
Left knee 2 months after intervention			
A	2.9 ± 0.7		
B	2.9 ± 0.3		
**Physical function score**		1509.1	0.0001
Right knee before intervention			
A	44.6 ± 4.4		
B	47.3 ± 7.2		
Left knee before intervention			
A	44.5 ± 4.3		
B	47.4 ± 7.1		
Right knee 1 month after intervention			
A	35.7 ± 3.7		
B	38.2 ± 5.6		
Left knee 1 month after intervention			
A	35.4 ± 4.1		
B	38.3 ± 5.5		
Right knee 2 months after intervention			
A	26.6 ± 4.2		
B	27.6 ± 5.2		
Left knee 2 months after intervention			
A	25.7 ± 2.5		
B	27.1 ± 5.2		
**WOAMC score**		1886	0.0001
Right knee before intervention			
A	64.7 ± 5.7		
B	67.6 ± 9.7		
Left knee before intervention			
A	64.7 ± 6.0		
B	67.8 ± 9.5		
Right knee 1 month after intervention			
A	51.6 ± 4.3		
B	53.1 ± 7.3		
Left knee 1 month after intervention			
A	50.6 ± 4.9		
B	53.6 ± 7.1		
Right knee 2 months after intervention			
A	35.4 ± 2.5		
B	35.9 ± 6.1		
Left knee 2 months after intervention			
A	35.2 ± 3.3		
B	36.4 ± 6.2		

^a^Groups are compared using the one-way repeated measures ANOVA.

## 5. Discussion

The findings of this clinical trial on adults with knee osteoarthritis suggest that hypertonic dextrose Prolotherapy in the peri-articular areas can effectively reduce pain, lower joint stiffness, and improve physical performance.

In a study conducted by Rezasoltani et al., 104 patients with knee osteoarthritis were divided into two groups. Injections of dextrose were administered intra-articularly to one group and peri-articularly to the other at four intervals. Their study's findings demonstrated that peri-articular injections outperformed intra-articular injections in terms of reducing knee disability and WOMAC score. The researchers combined 5 mL of 1% lidocaine with 5 mL of 20% dextrose for peri-articular injections and injected 2.5 mL of the solution subcutaneously at four points corresponding to acupuncture points around the knee where the periarticular nerves exit the joint capsule (9).

Our study's results regarding the effectiveness of peri-articular prolotherapy on knee pain are consistent with the study of Rezasoltani et al. (9). However, the composition of our injected solution drug (8 mL of 10% dextrose and 2 mL of 2% lidocaine) and the site of injection (around the peri-articular ligaments and soft tissue attachments instead of just subcutaneously at acupuncture points) were different from that study.

Rabago et al. (13) compared the effect of dextrose prolotherapy with saline and physiotherapy on pain and WOMAC in 90 patients. Researchers injected saline and dextrose into two participant groups three times at intervals of one, nine, and fifteen days in a study. The patients received 22.5 mL of 15% dextrose subcutaneously at 15 points, and 0.5 mL of the solution was simultaneously injected at three bone-tendon junctions. The patients were followed for 52 weeks, and the maximum effect was observed after 26 weeks, which lasted until the end of the study.

Although the specifics of injection protocol and follow-up duration differed from our study, the general results regarding the effectiveness of peri-articular dextrose were consistent with our findings. This suggests that peri-articular dextrose injections can be an effective treatment for osteoarthritis pain.

Another study by Alyan and El-Rouby (14) compared the effects of perineural subcutaneous dextrose 5% injection with low-level laser therapy on osteoarthritis pain in 100 patients. Four sites on the body where the nerves leave the capsule were subcutaneously injected with 5% dextrose by the researchers. Consistent with our findings, the study's results demonstrated the drug's efficacy in lowering pain. However, we used a more concentrated dextrose (10%), and injected it around the peri-articular ligaments and soft tissue attachments instead of subcutaneously.

Farpour and Fereydooni (15) conducted a study on 52 patients with primary osteoarthritis, randomly divided into two groups. The patients underwent dextrose prolotherapy in two stages with an interval of two weeks. One group received 6 mL of intra-articular 25% dextrose, whereas the other received the same amount of medication in the three most sensitive peri-articular locations. The researchers examined the patients using the Oxford Knee Scale (OKS), WOMAC, and VAS in the 4th and 8th weeks after the last injection. The results showed that both intra-articular and peri-articular injections had a similar effect on the improvement of VAS, OKS, and WOMAC criteria (14). These findings are consistent with the results of our and previously mentioned studies, suggesting that dextrose injection can improve osteoarthritis evaluation indicators regardless of the injection site. In conclusion, this study provided valuable insights into the effectiveness of dextrose prolotherapy in treating primary osteoarthritis. The findings imply that peri-articular injections may significantly enhance patients' symptoms and quality of life. These results have substantial implications for osteoarthritis care, highlighting the potential advantages of dextrose injection as a non-surgical treatment. Dextrose injection was shown to safely alleviate chronic musculoskeletal pain and enhance function in various conditions, although the precise mechanism of action is not fully understood (16). In addition to the specific effects of dextrose, the trauma caused by the needle and the increase in local tissue volume may contribute to tissue-level effects (16). While more research is needed to fully understand the mechanisms of dextrose prolotherapy, it is a promising treatment option for osteoarthritis and other musculoskeletal conditions.

### 5.1. Limitations

This study faced certain limitations, such as a small sample size and a brief follow-up period. Despite the small sample size, the inclusion of patients with bilateral osteoarthritis was noteworthy, and the injection of study drugs to different points on two sides was sufficient to detect some differences between groups. However, this research has notable features such as using a randomized design and evaluating outcomes using three different measures. These strengths ensure that the results are reliable and valid and can be used to inform future research and clinical practice. In general, while there were some limitations to this study, the strengths outweighed them, making it a valuable contribution to the field of osteoarthritis research.

### 5.2. Conclusions

This research found that extra-articular dextrose injections may effectively decrease pain, enhance physical performance, and relieve morning stiffness, independent of injection location. Compared to other therapies, dextrose prolotherapy is a straightforward, safe, and cost-effective procedure that is easily accessible and devoid of problems for patients.

## Data Availability

The primary database for this study is available upon request to the corresponding author.

## Clinical Trial Registration Code:

This trial was registered in Iranian Registry of Clinical Trials under the clinical trial code IRCT20181114041657N1.
